# Carbothermal Synthesis of Sludge Biochar Supported Nanoscale Zero-Valent Iron for the Removal of Cd^2+^ and Cu^2+^: Preparation, Performance, and Safety Risks

**DOI:** 10.3390/ijerph192316041

**Published:** 2022-11-30

**Authors:** Yingying Shao, Chao Tian, Yanfeng Yang, Yanqiu Shao, Tao Zhang, Xinhua Shi, Weiyi Zhang, Ying Zhu

**Affiliations:** 1 Advanced Materials Institute, Shandong Engineering Research Centre of Municipal Sludge Disposal, Qilu University of Technology (Shandong Academy of Sciences), Jinan 250014, China; 2 Shandong Shanke Institute of Ecological Environment Co., Ltd., Jinan 250000, China

**Keywords:** carbonthermal reduction, nanoscale zero-valent iron, municipal sludge, heavy metals removal mechanism, environmentally friendly

## Abstract

The practical application of nanoscale zero-valent iron (NZVI) is restricted by its easy oxidation and aggregation. Here, sludge biochar (SB) was used as a carrier to stabilize NZVI for Cd^2+^ and Cu^2+^ removal. SB supported NZVI (SB-NZVI) was synthesized using the carbothermic method. The superior preparation conditions, structural characteristics, and performance and mechanisms of the SB-NZVI composites for the removal of Cd^2+^ and Cu^2+^ were investigated via batch experiments and characterization analysis. The optimal removal capacities of 55.94 mg/g for Cd^2+^ and 97.68 mg/g for Cu^2+^ were achieved at a Fe/sludge mass ratio of 1:4 and pyrolysis temperature of 900 °C. Batch experiments showed that the SB-NZVI (1:4-900) composite had an excellent elimination capacity over a broad pH range, and that weakly acidic to neutral solutions were optimal for removal. The XPS results indicated that the Cd^2+^ removal was mainly dependent on the adsorption and precipitation/coprecipitation, while reduction and adsorption were the mechanisms that play a decisive role in Cu^2+^ removal. The presence of Cd^2+^ had an opposite effect on the Cu^2+^ removal. Moreover, the SB-NZVI composites made of municipal sludge greatly reduces the leaching toxicity and bio-availability of heavy metals in the municipal sludge, which can be identified as an environmentally-friendly material.

## 1. Introduction

With the industrialization and fast development of the economy, a large amount of heavy metals have been released into the environment [1]. Heavy metals, including cadmium (Cd) and copper (Cu), frequently coexist in effluents produced by electroplating, mining, metal smelting, and other industrial activities [2,3]. The toxicity, bioenrichment, and nonbiodegradation of heavy metals pose serious threats to both animals and plants [4]. Cd is considered to be the most toxic heavy metal pollutant, and even low concentrations can cause significant health problems to humans and animals [5,6]. Overexposure to Cd has been reported in the literature to increase blood pressure and damage the bones and kidneys [7,8,9]. Itai–itai disease (“it hurts–it hurts disease”) was the name given to a mass Cd poisoning incident that resulted in large-scale kidney damage and osteomalacia in Toyama Prefecture, Japan, in 1912 [10,11]. Cu is essential for the development of tissues and bone and enzyme synthesis [12,13]. In addition, Cu is an essential nutrient for humans, animals, and plants. However, in excessive amounts, Cu can cause headache, queasiness, respiratory problems, chronic liver injury, and renal failure [14]. Hence, there is an urgent need to remove heavy metals from contaminated water to avoid negative impacts on the environment and human health.

Due to the highly reactive surface area and adsorption/reduction capacities, nanoscale zero-valent iron (NZVI) particles have been widely used for organic pollutants and heavy metals removal as a new kind of environmental functional material [15,16,17,18,19]. Research has shown that NZVI particles can remove pollutants via adsorption, reduction, precipitation, coprecipitation, and other mechanisms [18]. However, NZVI particles are easy to agglomerate and have poor stability when exposed to air, due to their relatively high surface energy and magnetic force [20]. Various carbon-based materials, such as activated carbon [21], graphene [22], and biochar [20] and types of clay including kaolin [23] and bentonite [24] have been used to prevent the aggregation and oxidation of NZVI.

Biochar, which is a solid substance formed by carbonization of carbon-rich biomass, has received increasing attention as an abundant low-cost solid waste feedstock, distinguished by an abundance of diverse functional groups and the large specific surface area [25]. The reduction and resource recovery of municipal sludge from wastewater treatment plants have continued to gain attention. Municipal sludge contains an abundance of organic matter and biomass as a suitable raw material for biochar preparation. Previous research has reported that sludge biochar can be applied in pollutant removal from waste water as an adsorptive or carrier material. The findings of Yan et al. [26] suggested that the maximal adsorption capacity of tetracycline of modified sludge biochar was 93.44 mg/g, while Yang et al. [27] reported that the capacity of sewage sludge biochar loaded with α-Fe_2_O_3_ and α-FeOOH to adsorb nickel was 35.50 mg/g. Magnetic sludge biochar synthesized by Ma et al. [25] showed favorable maximum adsorption capacities for tetracycline and ciprofloxacin of 145 and 74.2 mg/g, respectively.

NZVI is mainly prepared via liquid-phase reduction and carbothermal methods. However, because of the inherent limitations, the liquid-phase reduction method is only suitable for preparing small amounts of NZVI in the laboratory and is not suitable for large-scale production [28]. Hence, the carbothermal method is considered to have the greatest potential, as the preparation process is relatively simple and can achieve mass productivity. It has been reported that sucrose [29], activated carbon [30], mesoporous carbon [28], and lignin-derived biobased substances [31] are suitable carbon sources for the preparation of supported NZVI by the carbothermal method. To the best of our knowledge, although biochar derived from municipal sludge has been used for the removal of environmental pollutants, the combination of NZVI and municipal sludge-derived biochar has been rarely used for Cd^2+^ and Cu^2+^ removal from aqueous systems. Besides, municipal sludge contains a certain amount of heavy metals (Zn, Cu, Pb, Cr, Cd, etc.), and most of the heavy metals in the municipal sludge still remain in the sludge biochar supported NZVI (SB-NZVI) composite material during the preparation process. Therefore, the environmental safety risk of heavy metals in the application of SB-NZVI composite material in wastewater treatment must be considered.

In this study, municipal sludge was used as a carbon source to prepare NZVI by the carbothermal method. SB-NZVI composites were prepared and applied to the treatment of heavy metal wastewater. Cd^2+^ and Cu^2+^, as common heavy metal pollutants, were selected as the removal target of this experiment. The optimum preparation conditions were determined, and the removal efficiency and mechanism of heavy metals were investigated. The environmental safety risk of SB-NZVI composites in the treatment of heavy metal wastewater was analyzed by using the heavy metal leaching test and the European Community Bureau of Reference (BCR) sequential extraction technique. The study provides a new method for resource utilization of municipal sludge.

## 2. Materials and Methods

### 2.1. Materials

The municipal sludge used in our research was obtained from the Qingdao Loushanhe Sewage Treatment Plant (Qingdao, China). All of the chemicals used in this study were of analytical grade. Ferric nitrate nonahydrate (Fe(NO_3_)_3_·9H_2_O), cupric chloride dihydrate (CuCl_2_·2H_2_O), and cadmium chloride (CdCl_2_·2.5H_2_O) were purchased from Tianjin Kemiou Chemical Reagent Co., Ltd. (Tianjin, China). The water used in this research was deionized water (Millipore Corporation, 18.2 MΩ).

### 2.2. Preparation of Materials

Municipal sludge was dried at 100 °C and filtered through 80–100 mesh. Municipal sludge biochar was prepared in a tube furnace by heating municipal sludge at 5 °C/min to one of four alternative temperatures (600, 700, 800, or 900 °C), followed by continuous pyrolyzation for 2 h until complete carbonization. Before heating, the tube was continuously filled with nitrogen for 45 min to remove oxygen. 

For the preparation of the SB-NZVI composites, Fe(NO_3_)_3_·9H_2_O and sludge were thoroughly stirred in deionized water at Fe/sludge mass ratios of 1:2, 1:4, 1:6, and 1:8, and dried at 60 °C. Then, the obtained samples were placed in a porcelain boat and heated in a tube furnace followed by pyrolyzation at one of four alternative temperatures (600, 700, 800, or 900 °C). Finally, the obtained composite materials were cooled to room temperature and then stored in nitrogen-filled glass bottles. The composite materials were denoted as SB-NZVI(1:n-T) for convenience, where “T” is the carbonization temperature and “1:n” is the Fe/sludge mass ratio.

### 2.3. Characterization of SB and SB-NZVI

Scanning Electron Microscopy (SEM; ZEISS MERLIN Compact, Germany) was used to assess the surface morphology of the SB-NZVI particles. X-ray diffraction (XRD; Ultima IV; Rigaku Corporation, Tokyo, Japan) was used to assess the structure and composition of the SB-NZVI particles. X-ray photoelectron spectrometry (XPS; K-Alpha; Thermo Fisher Scientific, Waltham, MA, USA) was used to determine the element composition and the valence state on the surfaces of the BC-NZVI particles before and after reacting with the pollutants.

### 2.4. Batch Removal Experiments

Batch heavy metal removal experiments were implemented in triplicate and average values were used for analysis. First, 0.025 g of material was added into 50 mL of reaction solutions in 100 mL conical flasks at 150 rpm and 25 °C. The suspensions were collected, filtered through a filter with 0.45 μm pores, and measured with an atomic absorption spectrometer (iCE™ 3000 Series; Thermo Fisher Scientific, Waltham, MA, USA). 

#### 2.4.1. Single Heavy Metal Removal

The ability to remove heavy metals from solution by different synthesized materials was investigated. The temperature was set from 600 °C to 900 °C to determine the optimum temperature for the preparation of the SB-NZVI composites. 

A temperature of 900 °C was chosen to prepare the SB-NZVI composites for heavy metal removal at Fe/sludge mass ratios of 1:2, 1:4, 1:6, and 1:8. The effect of the Fe/sludge mass ratio was investigated using the following pseudo-first-order and pseudo-second-order models (Equations (1) and (2), respectively).

(1) $$\ln\left(q_{e}-q_{t}\right)=lnq_{e}-k_{1}\cdot t$$

(2) $$t/q_{t}=1/k_{2}q_{e}^{2}+t/q_{e}$$

where *q_e_* and *q_t_* (mg/g) represent the amounts of heavy metals removed at equilibrium, *t* is time (min), and *k*_1_ (1/h) and *k*_2_ (g/mg·h) are the reaction rate constants.

Additional experiments were conducted to determine the optimal pH value of the solutions. The initial pH values of the separate heavy metal solutions ranged from 3.0 to 6.0 and 3.0 to 8.0, respectively. HCl and NaOH solutions (0.01 and 0.1 mol/L) were used to adjust the solution pH and were measured at specified time intervals (in the range of 5–60 min). 

The removal efficiency (*RE*, %) and removal capacity (*q_t_*, mg/g) were calculated as follows:

(3) $$RE=\left(C_{0}-C_{t}\right)/C_{0}\times 100\%$$

(4) $$q_{t}=\left(C_{0}-C_{t}\right)V/m$$

where *C*_0_ and *C_t_* (mg/L) represent the original and instantaneous concentrations at time *t*, respectively. *V* (L) is the volume of heavy metals solution, and *m* (g) is the amount of the added SB or SB-NZVI.

#### 2.4.2. Simultaneous Removal of Heavy Metals

Seven different experimental set-ups with different combinations of Cd^2+^ and Cu^2+^ were used to study the removal performance of SB-NZVI(1:4-900). The detail experimental was conducted as follows: when the initial concentration of Cd^2+^ was fixed at 30 mg/L, the initial Cu^2+^ concentrations were set at specified concentration intervals (i.e., 0, 1, 5, 10, 20, 30, and 50 mg/L), in addition, fixed the initial concentration of Cu^2+^ at 50 mg/L, changed the initial Cd^2+^ concentration at one of seven alternative concentrations (i.e., 0, 1, 5, 10, 15, 20, and 30 mg/L).

### 2.5. Environmental Safety Risk Analysis

#### 2.5.1. Speciation Analysis of Heavy Metals

The speciation of heavy metals was analyzed by using the BCR sequential extraction technique [32]. According to the BCR sequential extraction technique, the heavy metal forms can be divided into four species: the acid-soluble/exchangeable state (F1), the reducible state (F2), the oxidizable state (F3), and the residual state (F4).

The speciation of heavy metals in NZVI-SB composites determines its eco-toxicity. The residual state (F4) is considered as a stable state with less environmental toxicity. The acid-soluble/exchangeable state (F1) and the reducible state (F2) are considered as unstable states, which have a higher environmental toxicity. The oxidizable state (F3) is the potential effective state, and the environmental toxicity is relatively weak.

#### 2.5.2. Heavy Metal Leaching Test

A leaching test is a process that determines whether the contaminants are transferred from a solid to liquid medium. Therefore, the leaching test plays a vital role in assessing the environmental safety. The toxicity characteristic leaching procedure (TCLP) test is one of the most widely used ecological risk assessment methods in the world. In this study, TCLP test was applied to evaluate the leaching toxicity of heavy metals in civil sludge, SB and SB-NZVI.

In the TCLP toxic leaching experiment, extraction agents with different pH values were selected according to the sample’s pH and buffering capacity: when the pH was less than 5.0, we chose the extraction agent 1 (pH = 4.93 ± 0.05), when the pH was above 5, chose the extraction agent 2 (pH = 2.88 ± 0.05).

According to the pH and buffering capacity of the samples, extraction agent 2 was used in the present experiment. Then, 100 g of sample was placed in the extraction bottle and 2000 mL of extraction agent 2 (add 5.7 mL of glacial acetic acid in distilled water and then fixed the volume in 1 L, the pH was 2.88 ± 0.05 if correctly prepared) was added to achieve a solid/liquid ratio of 1:20. After the cap was closed, the bottle was fixed on the rotating oscillation device, and shaken at a speed of 30 ± 2 r/min for 18 ± 2 h at room temperature. After the shock, the extraction agent was passed through a 0.45 µm filter membrane and then the concentration of heavy metals was analyzed by the atomic absorption spectrometer (AAS).

## 3. Results and Discussion

### 3.1. Characterization of SB and SB-NZVI

#### 3.1.1. XRD Analysis

**Figure 1:**
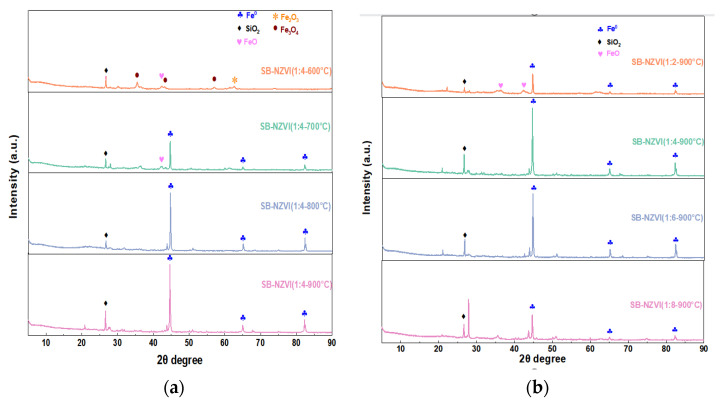
XRD patterns of (**a**) SB-NZVI at different temperatures and (**b**) with different mass ratios of Fe to sludge.

The XRD patterns of the SB-NZVI composites are exhibited in Figure 1. As shown in Figure 1a, the composites exhibited peaks of Fe_3_O_4_ (JCPDS No. 19-0629) at 2θ of 35.5°, 43.2°, and 57° at a pyrolysis temperature of 600 °C [33], indicating that the obtained composites were SB-Fe_3_O_4_. At a pyrolysis temperature of 700 °C, diffraction peaks at 44.7°, 65.2°, and 82.3° representing Fe^0^ (JCPDS No. 06-0696) emerged, while trace amounts of FeO (JCPDS No. 06-0615) still appeared, which implied that greatest amount of Fe_3_O_4_ was reduced to Fe^0^ by carbothermal reduction. At pyrolysis temperatures of 800 and 900 °C, the diffraction peak of Fe_3_O_4_ disappeared, indicating that the Fe_3_O_4_ was completely converted to Fe^0^. In the subsequent heavy metal removal experiments by composite materials prepared at different temperatures, the results showed that the higher the preparation temperature, the better the removal capacity (Figure S1). Thus, the optimum temperature was identified at 900 °C for the synthesis of SB-NZVI composites. The XRD patterns of the SB-NZVI composites prepared with different Fe/sludge mass ratios are shown in Figure 1b. At a Fe/sludge mass ratio of 1:2, FeO coexisted with Fe^0^ in the composites, when the Fe/sludge mass ratio increased to 1:4, the FeO peaks gradually decreased. This phenomenon indicates that a sufficient carbon source is favorable for the generation of Fe^0^. With the continuous increase in Fe/sludge mass ratio (1:6 to 1:8), the intensity of the Fe^0^ characteristic peak gradually decreased, which is the manifestation of the decrease in the Fe^0^ proportion in the composite.

The XRD analysis indicated that the carbothermal reduction method for the preparation of Fe^0^ required a specific temperature and a sufficient source of carbon. With the increase in temperature and a sufficient carbon source, Fe_3_O_4_ was reduced to FeO and then to Fe^0^. The mechanism of the carbothermal reduction method for preparing Fe^0^ can be expressed as follows, as (5) and (6) [31,34]:

(5) $${\mathrm{Fe}}_{3}O_{4}+0.5C\to 3\mathrm{FeO}+0.5{\mathrm{CO}}_{2}$$

(6) $$\mathrm{FeO}+0.5C\to {\mathrm{Fe}}^{0}+0.5{\mathrm{CO}}_{2}$$

#### 3.1.2. SEM Analysis

**Figure 2:**
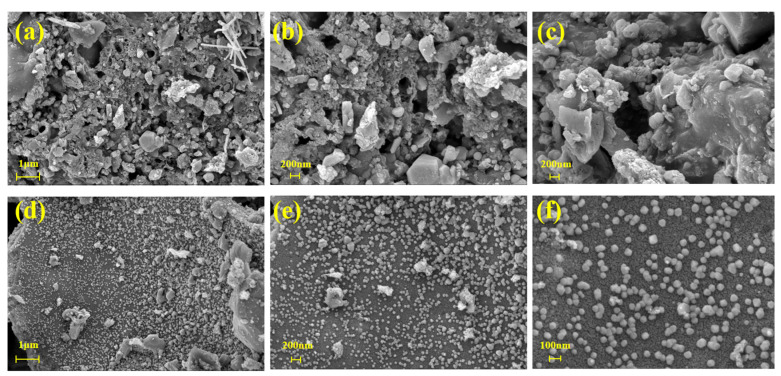
SEM images of (**a**–**c**) SB and (**d**–**f**) SB-NZVI(1:4-900).

**Figure 3:**
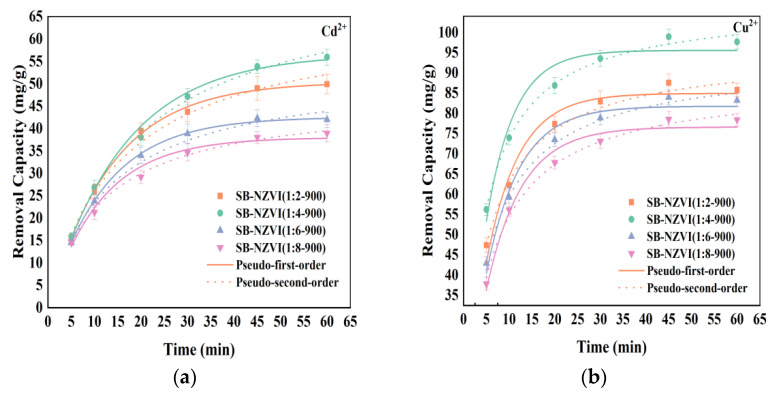
Adsorption kinetics models fitting for (**a**) Cd^2+^ and (**b**) Cu^2+^ removal by SB-NZVI composites at different mass ratios.

The size of the NZVI component in the SB-NZVI(1:4-900) composites and the surface morphology of the SB component at a pyrolysis temperature of 900 °C were observed by SEM, and the results are presented in Figure 2. It can be seen in Figure 2a and Figure 3c that SB had abundant folds with a rough and porous surface. During the pyrolysis process, the organic matter in the sludge was decomposed due to the anaerobic conditions, while methane, carbon dioxide, and other gases were released, which benefited the formation of the porous structure. The structure of SB was suitable for NZVI loading. SEM images of the SB-NZVI(1:4-900) composites (Figure 2d–f) showed that the particles of NZVI were evenly dispersed on the SB surface with particle diameters of 40–100 nm. These results demonstrate that the NZVI particles of the SB-NZVI(1:4-900) composites were effectively dispersed.

### 3.2. The Effect of Preparation Temperature on Heavy Metals Removal

The removal capacities of the SB and SB-NZVI composites prepared at different temperatures were investigated. As shown in Figure S1, the preparation temperature had a notable influence on the heavy metal removal. The raw SB exhibited a poor removal capacity for Cd^2+^ and Cu^2+^, but a favorable removal of these two kinds of heavy metal ions was achieved by the SB-NZVI composites. This is abundant proof of the constructive effect of NZVI and the limited removal capacity of SB. Obviously, as the preparation temperature raised from 600 to 900 °C, the order of Cd^2+^ removal capacities by the SB-NZVI composites increased from 12.43 to 55.94 mg/g, and the removal capacity of the SB-NZVI(1:4-900) composite for the removal of Cu^2+^ were reached at 97.68 mg/g, a 62.55 mg/g increase compared with the removal capacity of the SB-NZVI(1:4-600) composite. 

It is remarkably, however, that SB-NZVI(1:4-900) displayed a more outstanding removal capacity although a pure Fe^0^ characteristic peak existed in both SB-NZVI(1:4-900) and SB-NZVI(1:4-800), as shown in the XRD analysis. This difference could probably be explained by the fact that SB-NZVI(1:4-800) was a transitional form of SB-NZVI(1:4-900), because the intensity of the Fe^0^ characteristic peak increased with the increase in the preparation temperature from 800 °C to 900 °C, as shown in Figure 1a. When the preparation temperature was 600 °C, the removal capacities of Cd^2+^ and Cu^2+^ were very low, and the iron in the composite existed in the form of Fe_3_O_4_. This can be interpreted as the iron in the form of Fe^0^ being more preferable to the removal of Cd^2+^ and Cu^2+^ compared with Fe_3_O_4_. These results indicated that NZVI prepared at 900 °C played a major role in the removal of heavy metals Cd^2+^ and Cu^2+^.

### 3.3. The Effect of Mass Ratios of Fe to Sludge on Heavy Metals Removal 

An adsorption kinetics model was used to explore the effect of the Fe/sludge mass ratio on the removal of Cd^2+^ and Cu^2+^. It can be seen in Figure 3, that the removal capacity was clearly influenced by the Fe/sludge mass ratio and the best performance of the SB-NZVI composites was achieved at a Fe/sludge mass ratio of 1:4. The Fe/sludge mass ratio mainly affected the degree of Fe_3_O_4_ reduction and the content of NZVI [33]. An increase in the Fe/sludge mass ratio from 1:2 to 1:4 increased the degree of Fe_3_O_4_ reduction with an increase in the carbon content, and the Cd^2+^ and Cu^2+^ removal capacity increased from 49.94 to 55.94 mg/g and 85.76 to 97.68 mg/g, respectively. Unfortunately, as the sludge content continued to increase, the capacities of the SB-NZVI(1:6-900) and SB-NZVI(1:8-900) composites for the removal of Cd^2+^ and Cu^2+^ were only 41.98 and 38.96 mg/g, and 83.23 and 78.40 mg/g, respectively. The decreased removal performance was mainly explained by the decrease in NZVI content in SB-NZVI(1:6-900) and SB-NZVI(1:8-900).

Furthermore, the rapid adsorption equilibrium of Cd^2+^ and Cu^2+^ was achieved by SB-NZVI composites within 60 min (Figure 3). The average percentage error (*APE*) was calculated to compare the suitability of the kinetic models and the fitting ability of the experimental data, and the calculation formula is as follows [35]:

(7) $$APE_{kinetic}\left(\%\right)=\frac{\sum \left|\left(q_{t,exp}-q_{t,cal}\right)/q_{t,exp}\right|}{N}\times 100$$

As seen from Table S1, the Cd^2+^ adsorption results were well fitted by the two adsorption kinetics models (R^2^ > 0.98). However, it can be found that the calculated APE values of pseudo-first-order model were lower than the values of the pseudo-second-order model, except the data of SB-NZVI(1:8-900). Thus, the results indicate that the pseudo-first-order model is more suitable to fit the Cd removal process for SB-NZVI composite materials. The higher equilibrium adsorption capacity (*q_e_*) of the pseudo first-order model revealed that the capacity of the SB-NZVI(1:4-900) composite to adsorb Cd^2+^ was greater than that of other composites synthesized from different Fe/sludge mass ratios. For Cu^2+^ removal, the pseudo second-order model had a higher correlation coefficient (*R*^2^) and lower APE, indicating that chemisorption was the rate limiting step during the reaction [36]. The corresponding reaction rate constant (*k*_2_) changed with the Fe/sludge mass ratio. This phenomenon is also evidence that the capacity of the SB-NZVI(1:4-900) to absorb Cu^2+^ was superior to that of other composites with different Fe/sludge mass ratios. Based on these results, the SB-NZVI(1:4-900) composite was selected for further study.

### 3.4. The Effect of Initial Solution pH on Heavy Metals Removal

**Figure 4:**
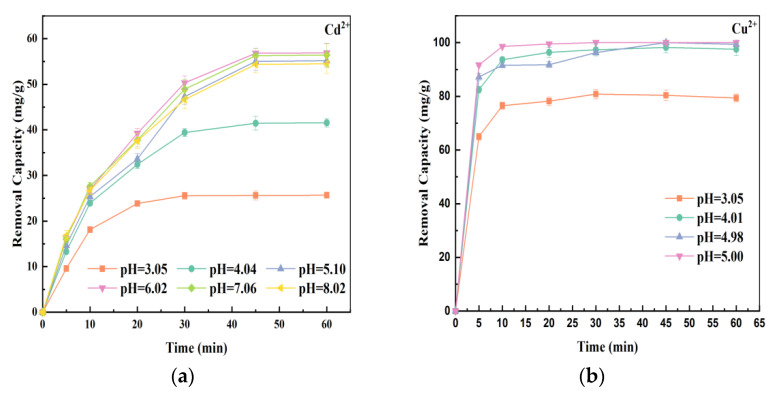

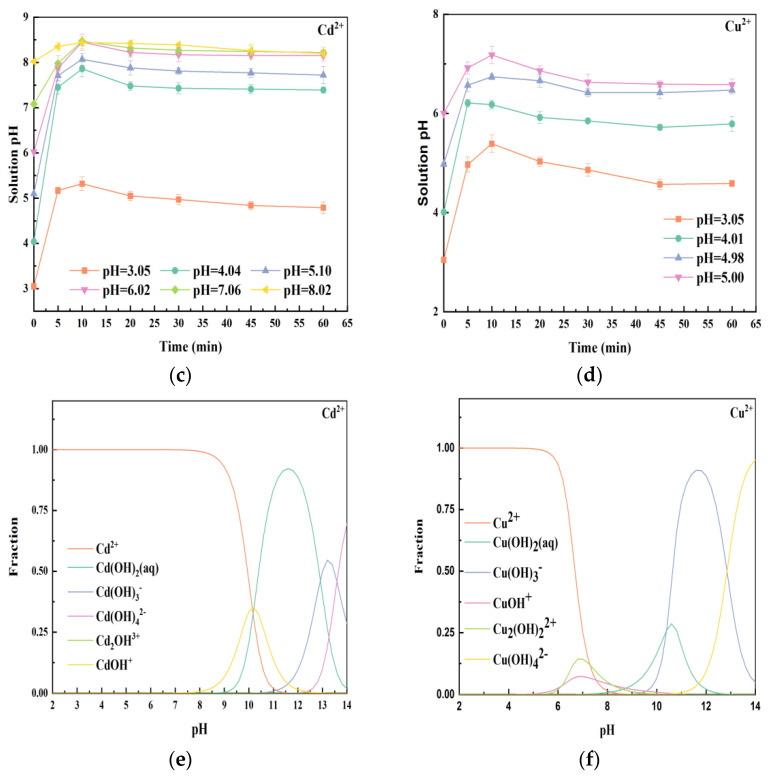
Effect of the initial solution pH on the (**a**) Cd^2+^ and (**c**) Cu^2+^ removal by SB-NZVI(1:4-900) composites; the change in solution pH during the reaction of (**b**) Cd^2+^ and (**d**) Cu^2+^ removal; the (**e**) Cd^2+^ and (**f**) Cu^2+^ species distribution as a function of pH.

Generally, the initial solution pH has a significant effect on the reaction system due to the influence on the degree of ionization, the distribution of pollutants, and the corrosion of iron particles [37,38]. The removal capacity of the composites for Cd^2+^ and Cu^2+^ was investigated at different initial pH values. To avoid the precipitation of heavy metals, the distribution of Cd^2+^ and Cu^2+^ was calculated with Visual MINTEQ 3.1 software. As shown in Figure 4e, at a pH value of <8.0, Cd^2+^ was the dominant species and Cd(OH)_2_ started to appear at about pH 9.6. Thus, a pH value of 3.00–8.02 was selected to avoid the precipitation of Cd^2+^. Similarly, Cu^2+^ was the dominant species at a pH value of <6, and Cu(OH)_2_ started to appear at about pH 6.2 (Figure 4f). Therefore, a pH value of 3.05–6.00 was selected for Cu^2+^ removal. Furthermore, the zeta potential values of SB-NZVI(1:4-900) were determined by Malvern Nano Zeta Sizer at different solution pH (from 4.0 to 9.0). The zero-point charge (pHpzc) of SB-NZVI(1:4-900) occurred at pH 5.85. 

The influence of different initial solution pH values on the Cd^2+^ removal capacity was studied at a set contact time (Figure 4a), while simultaneously measuring the pH variations of the solution during Cd^2+^ removal (Figure 4b). It was clear that the capacity of the composites to remove Cd^2+^ was distinctly affected by the initial solution pH. As illustrated in Figure 4a, the removal capacity increased from 25.67 to 56.88 mg/g as the initial pH increased from 3.05 to 6.02, but remained relatively stable at a pH range of 6.02–8.02. These results indicate that the SB-NZVI(1:4-900) composites had a good Cd^2+^ removal capacity (54.50–56.88 mg/g) over a wide pH range of 5.10–8.02. At an initial pH of 6.02, the maximum capacity of the SB-NZVI(1:4-900) composite to remove Cd^2+^ was 56.88 mg/g. Hence, the Cd^2+^ removal capacity of the SB-NZVI(1:4-900) composite was suitable in weakly acidic to neutral solutions.

The influence of the initial solution pH on Cu^2+^ removal was also investigated, while simultaneously measuring variations in the solution pH during Cu^2+^ removal (Figure 4d). As illustrated in Figure 4c, the capacity of the composites to remove Cu^2+^ at an initial pH of 3.05 was 79.38 mg/g, which was much lower than at other pH values. In comparison, the removal capacity remained stable as the pH of the solution changed from 4.01 to 6.00 (97.51–100.00 mg/g), suggesting an excellent removal capacity of the SB-NZVI(1:4-900) composite in acidic to neutral solutions.

These results can be explained by the following two scenarios: (1) at low pH values, the NZVI particles dissolved due to the high H^+^ concentration, which greatly reduced the adsorption and reduction capacity of NZVI, and (2) as described in previous studies [28,39], NZVI composites have a positive surface charge at low pH values (lower than 5.85 for the SB-NZVI(1:4-900) prepared in this study), leading to electrostatic repulsion to Cd^2+^ and Cu^2+^ ions. Hence, as the solution pH increased, the electrostatic repulsive forces were gradually weakened, which improved the capacity to remove Cd^2+^ and Cu^2+^. As illustrated in Figure 4b,d, the solution pH during removal of Cd^2+^ and Cu^2+^ were rapidly increased for the first 10 min and then gradually decreased with the reaction time. This phenomenon was most likely because of the OH^−^ ions produced by the reaction of NZVI and H_2_O. With the hydroxylation of Fe^2+^ ions, the solution pH steadily decreased to a state of equilibrium.

### 3.5. Simultaneous Removal of Cd^2+^ and Cu^2+^

Cd^2+^ and Cu^2+^ often coexist in effluents produced by electroplating, mining, metal smelting, and other industrial activities. Thus, the performance of the SB-NZVI(1:4-900) composite for the simultaneous removal of Cd^2+^ and Cu^2+^ was investigated at different concentration ratios of two heavy metals. As illustrated in Figure S2a, different initial concentrations of Cu^2+^ had an obvious influence on the removal efficiency of Cd^2+^, which was manifested as low Cu^2+^ initial concentrations (<5 mg/L), which promoted Cd^2+^ removal, and high Cu^2+^ initial concentrations (>5 mg/L), which inhibited Cd^2+^ removal. However, the removal efficiencies of different initial concentrations of Cu^2+^ were always above 97.5%. As shown in Figure S2b, different initial concentrations of Cd^2+^ had little effect on the removal of Cu^2+^, and the removal efficiencies of Cu^2+^ were always above 97.5%, while the removal efficiencies of Cd^2^+ decreased obviously with the increase in Cd^2+^ initial concentration. The simultaneous removal experiment results showed that the removal of Cu^2+^ was superior to that of Cd^2+^ removal, although the removal efficiency of both two heavy metals could be maintained at a high level (>85% under all simultaneous removal conditions).

### 3.6. Mechanisms for Heavy Metals Removal 

**Figure 5:**
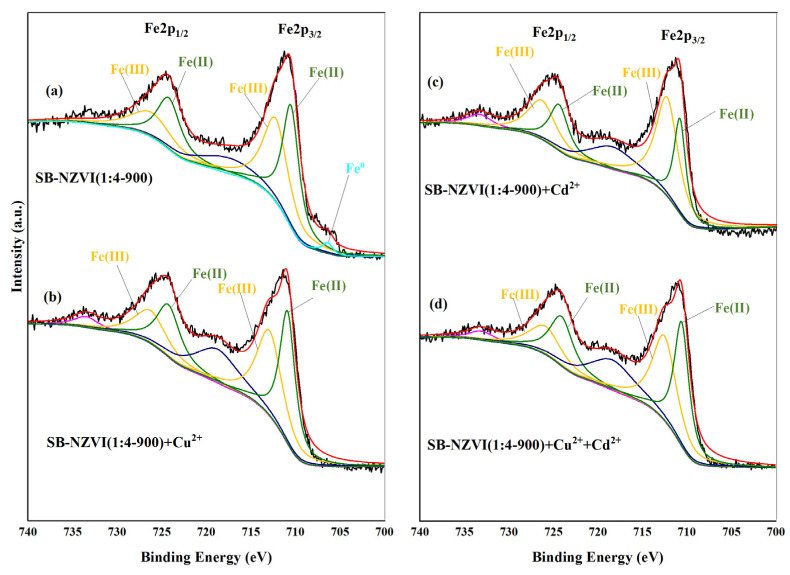
Fe 2p narrow scan spectra of SB-NZVI(1:4-900) (**a**) and after a reaction with Cu^2+^ (**b**), Cd^2+^ (**c**), and coexisting with two heavy metals (**d**).

**Figure 6:**
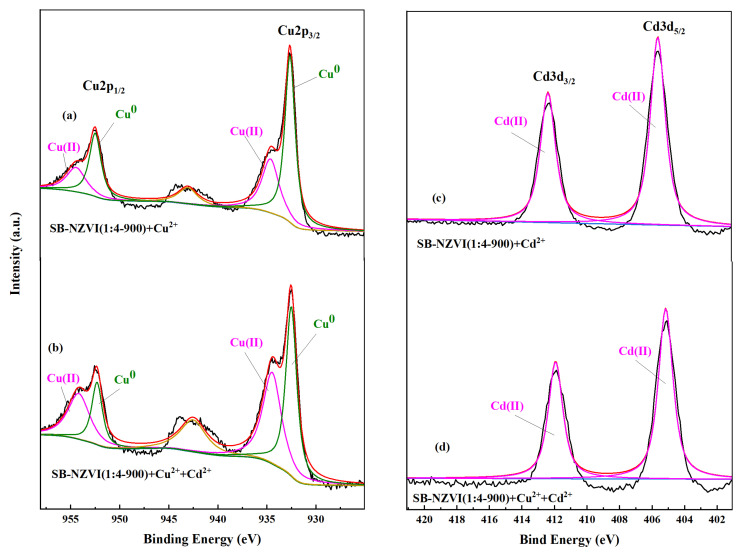
Cu 2p and Cd 3d narrow scan spectra of SB-NZVI(1:4-900) after reaction with (**a**) Cu^2+^, (**c**) Cd^2+^ and (**b**,**d**) coexisting with two heavy metals.

In order to investigate the mechanism underlying the capacity of the SB-NZVI(1:4-900) composite for the removal of Cd^2+^ and Cu^2+^, XPS analysis was used to measure the changes to the SB-NZVI(1:4-900) composite surface. The XPS results of the SB-NZVI(1:4-900) composite before and after the reaction are presented in Figure S3 and Figure 5 and Figure 6. The Fe2p spectrum of the raw SB-NZVI(1:4-900) composite (Figure 5a) contained a smaller peak at about 706.4 eV, which is attributed to Fe0, while the Fe2p3/2 peak at the binding energy of 710.5 eV and the Fe2p1/2 peak at the binding energy of 724.3 eV could be identified as Fe(Ⅱ), and the Fe2p3/2 peak at the binding energy of 712.2 eV and the Fe2p1/2 peak at the binding energy of 726.23 eV could be identified as Fe(Ⅲ) [40,41].

For Cd^2+^ removal, the XPS spectra of SB-NZVI(1:4-900)+Cd confirmed the presence of Cd in the solid material after the reaction (Figure S3), indicating that the SB-NZVI(1:4-900) composite achieved Cd^2+^ removal from the aqueous solution. As shown in Figure 5c, the characteristic peak of Fe^0^ at about 706.4 eV disappeared after the reaction with Cd^2+^ and the proportion of ferric iron to total iron (Fe(Ⅲ)/Fe(total)) increased from 0.48 to 0.62, indicating that Fe^0^ was oxidized after the reaction. A 3D narrow-scan spectrum of SB-NZVI(1:4-900) after the reaction with 30 mg/L of Cd^2+^ is displayed in Figure 6c. The corresponding Cd 3D peaks at 412.4 eV and 405.6 eV can be ascribed to Cd^2+^ species, confirming that the valence state of Cd did not change after the reaction with SB-NZVI(1:4-900). A reasonable explanation for this result is that because of the nearly identical standard potentials of Cd/Cd^2+^ (E_0_ = −0.40 V) and Fe/ Fe^2+^ (E_0_ = −0.41 V), reduction of Cd^2+^ by Fe^0^ was unlikely, suggesting that adsorption and precipitation/coprecipitation were the most probable mechanisms of the reaction, accompanied by the generation of Cd(OH)_2_ and Cd_x_Fe_(1−x)_(OH)_2_ [27,42,43]. 

The XPS spectra of SB-NZVI(1:4-900)+Cu in Figure S3 confirmed that the SB-NZVI(1:4-900) composites removed Cu^2+^ from the solution, similar to the conclusion of Cd^2+^ removal. As illustrated in Figure 5b, there was no Fe^0^ peak because the iron core was coated with iron oxide/hydroxide [27]. The Cu 2p narrow-scan spectra of SB-NZVI(1:4-900)+Cu is illustrated in Figure 6a. The peaks of binding energy at about 954.17 and 934.69 eV are characteristic of Cu^2+^, while those at around 952.31 and 932.51 eV are characteristic of Cu^0^/Cu_2_O [40,44]. The main Cu species were Cu^0^/Cu_2_O (63.88%) and Cu^2+^ (36.12%), which signified that the removal of Cu^2+^ by the SB-NZVI(1:4-900) composite mainly occurred by reduction, while adsorption played a relatively minor role. In addition, chemical precipitation played a certain role at a higher solution pH.

For simultaneous removal of two heavy metals at initial concentrations of 30 and 50 mg/L, the wide-scan XPS spectra of the SB-NZVI(1:4-900)+Cu+Cd composite confirmed the presence of Cu and Cd (Figure S3). The Cu 2p and Cd 3D narrow-scan spectra were similar to the spectra of the individual removal of two heavy metals, respectively (Figure 6). However, the primary Cu species in the binary removal system were Cu^0^/Cu_2_O (48.60%) and Cu^2+^ (51.40%), suggesting that a high concentration of Cd^2+^ inhibited the reduction process for Cu^2+^ removal. 

Based on these results, it can be concluded that different mechanisms were employed by the SB-NZVI(1:4-900) composite for the removal of two heavy metals. The removal of Cd^2+^ was mainly caused by adsorption and precipitation/coprecipitation, while the removal of Cu^2+^ was mainly due to reduction and adsorption.

### 3.7. Environmental Safety Risk Analysis of SB-NZVI

#### 3.7.1. Speciation Analysis of Heavy Metals

**Figure 7:**
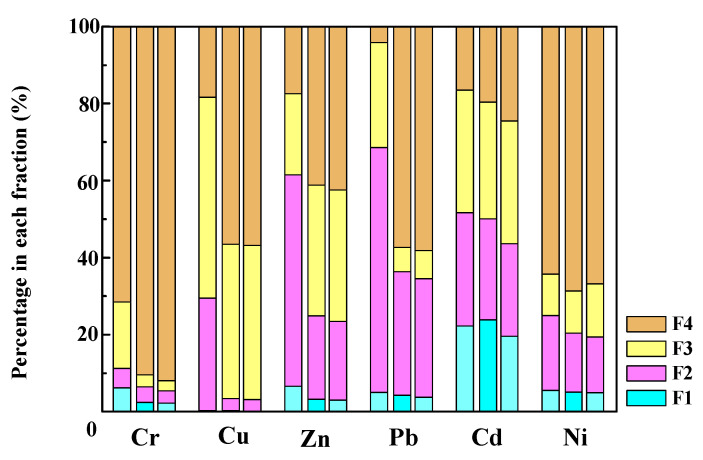
Heavy metal fraction of municipal sludge (left), SB (midial), and SB-NZVI (right).

The speciation of heavy metals in sludge, SB, and SB-NZVI varied greatly, as shown in Figure 7. In general, heavy metals in state of F1 and F2 have a stronger mobility and bioavailability according to their environmental conditions [45]. In the municipal sludge, heavy metals of Cd, Pb, and Zn were present at high percentages in the F1 and F2 state (51.71% for Cd, 68.56% for Pb, and 61.48% for Zn). The F3 state of Cu reached 52.13%, which may be due to the formation of more organic-Cu complexes [45,46]. Cr (71.52%) and Ni (64.26%) were concentrated in the F4 state. However, the contents of heavy metals of the F4 state in SB and SB-NZVI were significantly higher than the initial state distribution in municipal sludge. For instance, the amount of heavy metal Pb to F4 state increased significantly from 4.11% to 57.39% (SB) and 58.15% (SB-NZVI), respectively. Simultaneously, the F4 state of other heavy metals (Cr, Cu, Zn, Cd, and Ni) in SB and SB-NZVI increased compared with in municipal sludge. Therefore, pyrolysis can transform more heavy metals into residual state and fix them into SB and reduce its bioavailability, thereby reducing environmental risks.

#### 3.7.2. Heavy Metal Leaching Test

Table 1 shows the heavy metal leaching concentrations of sludge, SB, and SB-NZVI in the TCLP experiment. The leaching concentrations of heavy metals Cr, Cu, Zn, Pb, Cd, and Ni in the sludge were 0.78 mg/kg, 1.76 mg/kg, 16.96 mg/kg, 7.91 mg/kg, 0.68 mg/kg, and 3.66 mg/kg, respectively. After the municipal sludge was prepared into SB or SB-NZVI, the leaching concentration of heavy metals decreased to a great extent compared with the raw municipal sludge, indicating that the pyrolysis process effectively inhibited the leaching capacity of heavy metals in SB, which was consistent with the proportion of the F1 state of heavy metals in Figure 7.

Above all, the speciation analysis of heavy metals and heavy metal leaching test indicated that the SB-NZVI composite is an environmental-friendly material and poses less of a threat to water resources in the wastewater treatment process.

## 4. Conclusions

In the present study, an SB-NZVI(1:4-900) composite was synthesized by the carbothermal reduction method as an efficient functional material for Cd^2+^ and Cu^2+^ removal from single and binary aqueous systems. Batch experiments indicated that the SB-NZVI(1:4-900) composite effectively achieved Cd^2+^ and Cu^2+^ removal from aqueous systems. The SB component effectively prevented the aggregation of the NZVI particles, which enhanced the removal capacity of the SB-NZVI(1:4-900) composite. The XRD, SEM, and XPS results confirmed that the NZVI particles (40–100 nm) were dispersed on the SB surface. The SB-NZVI(1:4-900) composite could remove 55.94 mg/g of Cd^2+^ and 97.68 mg/g of Cu^2+^ within 60 min, and a weakly acidic to neutral solution promoted the removal of heavy metals through the SB-NZVI(1:4-900) composite. Adsorption and precipitation/coprecipitation were the main mechanisms responsible for Cd^2+^ removal, while Cu^2+^ was primarily removed by reduction and adsorption. Lastly, the presence of Cd^2+^ was detrimental to the removal of Cu^2+^. The results of the environmental safety risk analysis show that the SB-NZVI composite is an environmental-friendly material and is less of a threat to water resources in the wastewater treatment process.

## Figures and Tables

**Table 1:** Leaching concentration of heavy metals from municipal sludge, SB, and SB-NZVI.

Samples	Heavy Metals (mg/kg)					
	Cr	Cu	Zn	Pb	Cd	Ni
sludge	0.78 ± 0.01	1.76 ± 0.12	16.96 ± 0.61	7.91 ± 0.25	0.68 ± 0.05	3.66 ± 0.09
SB	0.02 ± 0.01	0.25 ± 0.05	6.36 ± 0.25	7.26 ± 0.37	0.71 ± 0.02	2.88 ± 0.14
SB-NZVI	ND	0.17 ± 0.04	5.68 ± 0.08	6.93 ± 0.19	0.62 ± 0.03	2.71 ± 0.14

## Data Availability

The data presented in this study are available upon request from the corresponding author.

## Supplementary Materials

The following are available online at https://www.mdpi.com/article/10.3390/ijerph192316041/s1. Table S1: Adsorption kinetics of SB-NZVI composites for Cd^2+^ and Cu^2+^ removal. Figure S1: Effect of preparation temperature on the Cd^2+^ and Cu^2+^ removal by SB and SB-NZVI composites, Figure S2: Effect of (a) different Cu^2+^ initial concentration on the removal of Cd^2+^ and (b) different Cd^2+^ initial concentration on the removal of Cu^2+^ by SB-NZVI(1:4-900), Figure S3: XPS Wide-scan spectra of SB-NZVI(1:4-900) and after reaction with Cd^2+^, Cu^2+^ and coexisting Cd^2+^ and Cu^2+^.
