# Supplementary material for: Carbothermal Synthesis of Sludge Biochar Supported Nanoscale Zero-Valent Iron for the Removal of Cd2+ and Cu2+: Preparation, Performance, and Safety Risks
Source: Int J Environ Res Public Health. 2022 Nov 30;19(23):16041. doi: 10.3390/ijerph192316041 (PMC9740856; doi:10.3390/ijerph192316041)
Supplement: Supplementary file 1 [file ijerph-19-16041-s001.zip › ijerph-1997243-supplementary.pdf]

Supplementary Material for:

Carbothermal Synthesis of Sludge Biochar Supported Nanoscale Zero-Valent Iron for the Removal of Cd^{2+} and Cu^{2+}: Preparation, Performance and Safety Risks

Yingying Shao [1,2], Chao Tian [2], Yanfeng Yang [2], Yanqiu Shao [1], Tao Zhang [1], Xinhua Shi [1], Weiyi Zhang [1,*] and Ying Zhu[1,*]

[1] Qilu University of Technology (Shandong Academy of Sciences), Advanced Materials Institute, Shandong Engineering Research Centre of Municipal Sludge Disposal, Jinan 250014, China

[2] Shandong Shanke Institute of Ecological Environment Co. LTD, Jinan 250000, China

* Correspondence: zhangweiyi@sdas.org (W.Z.); zhuyingee@163.com (Y.Z.); Tel.: +86-0531-82605428 (W.Z.); +86-0531-82605428 (Y.Z.)

Table S1 Adsorption kinetics of SB-NZVI composites for Cd^{2+} and Cu^{2+} removal

Adsorbents	Metal ions	Pseudo-first-order model				Pseudo-second-order model			
		k_1(1/h)	*q_e(mg/g)*	*R^2*	*APE(%)*	*k_2(g/(mg.h))*	*q_e(mg/g)*	*R^2*	*APE(%)*
SB-NZVI（1:2-900）	Cd^{2+}	0.07354	50.42022	0.99891	1.102	0.001	65.44117	0.99155	2.735
	Cu^{2+}	0.14166	84.93436	0.96148	3.469	0.00207	95.14273	0.98824	1.706
SB-NZVI（1:4-900）	Cd^{2+}	0.06282	56.67829	0.9958	1.709	0.00074	74.31772	0.99602	1.838
	Cu^{2+}	0.16329	95.57288	0.95591	3.661	0.00209	106.82644	0.99523	0.864
SB-NZVI（1:6-900）	Cd^{2+}	0.08281	42.55835	0.99917	0.966	0.00148	53.0993	0.99152	3.258
	Cu^{2+}	0.13657	81.77104	0.97459	3.198	0.00189	93.08899	0.99336	1.279
SB-NZVI（1:8-900）	Cd^{2+}	0.08831	38.01006	0.98043	4.503	0.00188	46.95089	0.99665	2.066
	Cu^{2+}	0.12893	76.62008	0.97818	2.816	0.00185	87.96164	0.99117	1.904

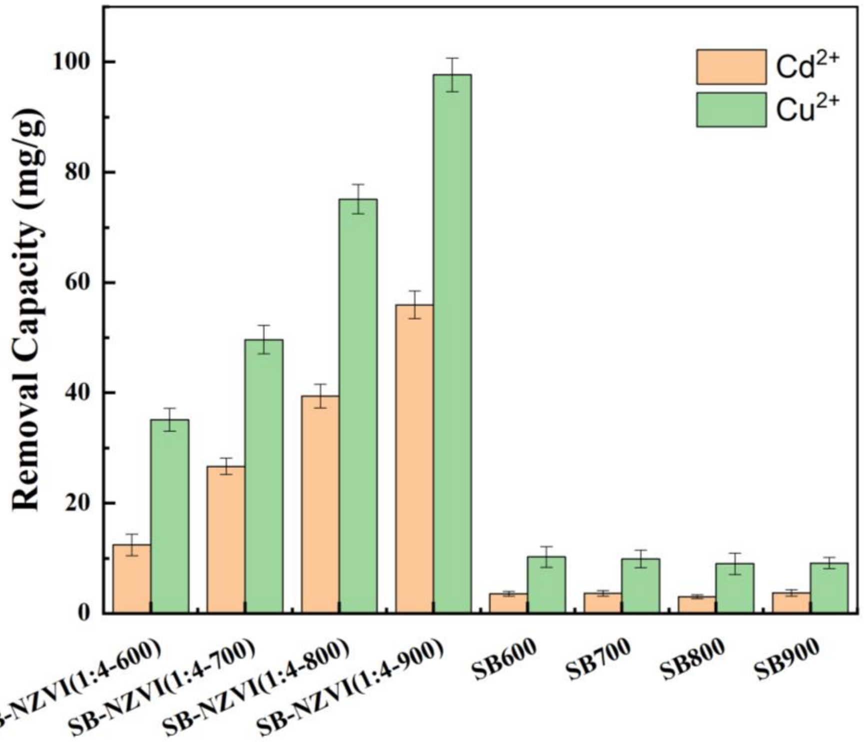

Figure S1. Effect of preparation temperature on the Cd^{2+} and Cu^{2+} removal by SB and SB-NZVI composites.

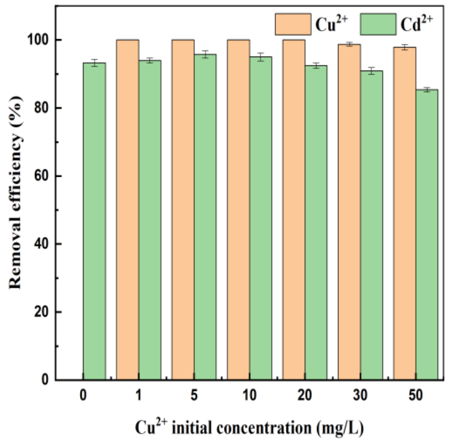

(**a**)

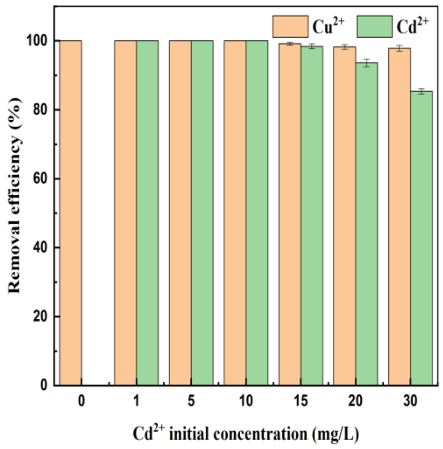

(**b**)

Figure S2. Effect of (a) different Cu^{2+} initial concentration on the removal of Cd^{2+} and (b) different Cd^{2+} initial concentration on the removal of Cu^{2+} by SB-NZVI(1:4-900)

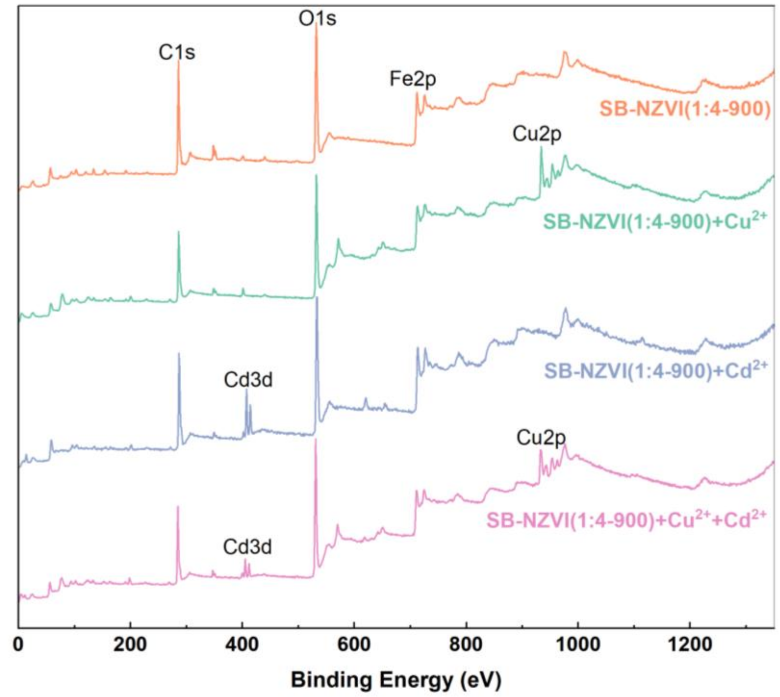

Figure S3. XPS Wide-scan spectra of SB-NZVI(1:4-900) and after reaction with Cd^{2+}, Cu^{2+} and coexisting Cd^{2+} and Cu^{2+}
